# Glioma Grade and Molecular Markers: Comparing Machine-Learning Approaches Using VASARI (Visually AcceSAble Rembrandt Images) Radiological Assessment

**DOI:** 10.7759/cureus.63873

**Published:** 2024-07-04

**Authors:** Nurhuda H Setyawan, Lina Choridah, Hanung A Nugroho, Rusdy G Malueka, Ery K Dwianingsih, Yana Supriatna, Bambang Supriyadi, Rachmat A Hartanto

**Affiliations:** 1 Department of Radiology, Faculty of Medicine, Public Health, and Nursing, Dr. Sardjito General Hospital, Universitas Gadjah Mada, Yogyakarta, IDN; 2 Department of Electrical and Information Engineering, Faculty of Engineering, Universitas Gadjah Mada, Yogyakarta, IDN; 3 Department of Neurology, Faculty of Medicine, Public Health, and Nursing, Dr. Sardjito General Hospital, Universitas Gadjah Mada, Yogyakarta, IDN; 4 Department of Anatomical Pathology, Faculty of Medicine, Public Health, and Nursing, Dr. Sardjito General Hospital, Universitas Gadjah Mada, Yogyakarta, IDN; 5 Department of Radiology, Faculty of Medicine, Public Health, and Nursing,Dr. Sardjito General Hospital, Universitas Gadjah Mada, Yogyakarta, IDN; 6 Department of Surgery, Faculty of Medicine, Public Health, and Nursing, Dr. Sardjito General Hospital, Universitas Gadjah Mada, Yogyakarta, IDN

**Keywords:** mgmt methylation, idh mutation status, machine learning, vasari radiological features, gliomas

## Abstract

Objectives: This study aimed to leverage Visually AcceSAble Rembrandt Images (VASARI) radiological features, extracted from magnetic resonance imaging (MRI) scans, and machine-learning techniques to predict glioma grade, isocitrate dehydrogenase (IDH) mutation status, and O6-methylguanine-DNA methyltransferase (MGMT) methylation.

Methodology: A retrospective evaluation was undertaken, analyzing MRI and molecular data from 107 glioma patients treated at a tertiary hospital. Patients underwent MRI scans using established protocols and were evaluated based on VASARI criteria. Tissue samples were assessed for glioma grade and underwent molecular testing for IDH mutations and MGMT methylation. Four machine learning models, namely, Random Forest, Elastic-Net, multivariate adaptive regression spline (MARS), and eXtreme Gradient Boosting (XGBoost), were trained on 27 VASARI features using fivefold internal cross-validation. The models' predictive performances were assessed using the area under the curve (AUC), sensitivity, and specificity.

Results: For glioma grade prediction, XGBoost exhibited the highest AUC (0.978), sensitivity (0.879), and specificity (0.964), with f6 (proportion of non-enhancing) and f12 (definition of enhancing margin) as the most important predictors. In predicting IDH mutation status, XGBoost achieved an AUC of 0.806, sensitivity of 0.364, and specificity of 0.880, with f1 (tumor location), f12, and f30 (perpendicular diameter to f29) as primary predictors. For MGMT methylation, XGBoost displayed an AUC of 0.580, sensitivity of 0.372, and specificity of 0.759, highlighting f29 (longest diameter) as the key predictor.

Conclusions: This study underscores the robust potential of combining VASARI radiological features with machine learning models in predicting glioma grade, IDH mutation status, and MGMT methylation. The best and most balanced performance was achieved using the XGBoost model. While the prediction of glioma grade showed promising results, the sensitivity in discerning IDH mutations and MGMT methylation still leaves room for improvement. Follow-up studies with larger datasets and more advanced artificial intelligence techniques can further refine our understanding and management of gliomas.

## Introduction

Gliomas are a group of tumors originating from the central nervous system's glial cells, and they represent approximately 30% of all brain and central nervous system tumors and 80% of all malignant brain tumors. The global burden of gliomas is considerable, with an estimated incidence rate of 6 per 100,000 people worldwide [1]. Glioblastoma, the most aggressive form, has a particularly poor prognosis, with a median survival time of less than 15 months despite multimodal treatments including surgery, radiation, and chemotherapy [2].

The 2021 World Health Organization (WHO) classification of tumors of the central nervous system has emphasized the integration of molecular parameters alongside histological features for more accurate and prognostically meaningful categorization of gliomas. Molecular markers like isocitrate dehydrogenase (IDH) mutations and O6-methylguanine-DNA methyltransferase (MGMT) promoter methylation status have gained substantial attention for their role in tumor classification, prognosis, and treatment planning. IDH mutations are associated with a better prognosis and are often found in lower-grade gliomas and secondary glioblastomas. MGMT promoter methylation is indicative of a better response to alkylating agent chemotherapy, such as temozolomide, and is correlated with improved overall survival [3].

Traditional methods of evaluating gliomas, such as histopathological analysis, are invasive and subject to sampling bias [4]. Hence, there is a growing interest in the development of noninvasive techniques to characterize these tumors effectively. One such approach involves the use of VASARI (Visually AcceSAble Rembrandt Images) features, which are standardized radiological attributes extracted from magnetic resonance imaging (MRI) scans. The VASARI features are a standardized set of 27 radiological attributes used to characterize gliomas based on MRI scans. These features include aspects such as tumor location, dimensions, shape, patterns of enhancement, necrosis, and edema. For example, feature f1 represents the location of the tumor, feature f6 denotes the proportion of the tumor that is non-enhancing, and feature f12 describes the definition of the enhancing margin. These detailed radiological features provide a comprehensive assessment of the tumor's morphology and are crucial for the subsequent machine-learning analysis [5,6].

Machine learning techniques offer a promising avenue for furthering our understanding of gliomas. Previous studies have employed machine learning algorithms to predict glioma grades and molecular markers like IDH mutation status and MGMT methylation [7,8]. These predictive models integrate radiological features with clinical and molecular data, enhancing their ability to inform clinical decisions [6]. Despite these advances, several gaps in the literature persist. For instance, most studies focus on individual machine learning models and a limited set of VASARI features. Additionally, there is a need to examine the performance of these predictive models in terms of their sensitivity and specificity, as well as their applicability in diverse patient populations.

The present study aims to fill these gaps by employing multiple machine learning models-Random Forest, Elastic-Net, multivariate adaptive regression spline (MARS), and eXtreme Gradient Boosting (XGBoost)-to predict glioma grades and key molecular markers using 27 VASARI features. Elastic-Net is a regularization technique that linearly combines the L1 and L2 penalties of the Lasso and Ridge methods to enhance model performance, especially when dealing with highly correlated variables. MARS is a nonparametric regression technique that builds flexible models by fitting piecewise linear regressions, which are particularly useful for capturing non-linear relationships in the data. Random Forest is an ensemble learning method that constructs multiple decision trees during training and outputs the mode of the classes (classification) or mean prediction (regression) of the individual trees, providing improved accuracy and robustness. XGBoost is an optimized gradient-boosting framework that builds an ensemble of decision trees sequentially, optimizing for prediction accuracy and incorporating regularization to prevent overfitting.

We aim to assess the inter-observer and intra-observer consistency in VASARI feature extraction. We also evaluate the performance of multiple machine learning models in predicting glioma grade, IDH mutation status, and MGMT methylation, as well as identify which VASARI features hold the most predictive power in these models. By addressing these research questions, this study seeks to contribute to the existing body of knowledge on the utility of machine learning and VASARI features in glioma characterization and to inform future research and clinical practices.

## Materials and methods

Ethical approval

All datasets were de-identified and incorporated into this research per an approved retrospective protocol by the Institutional Review Board (IRB) at Universitas Gadjah Mada. The IRB approved the number KE/FK/1182/EC/2022, and written consent was obtained from all participating individuals.

Subject selection

**Figure 1:**
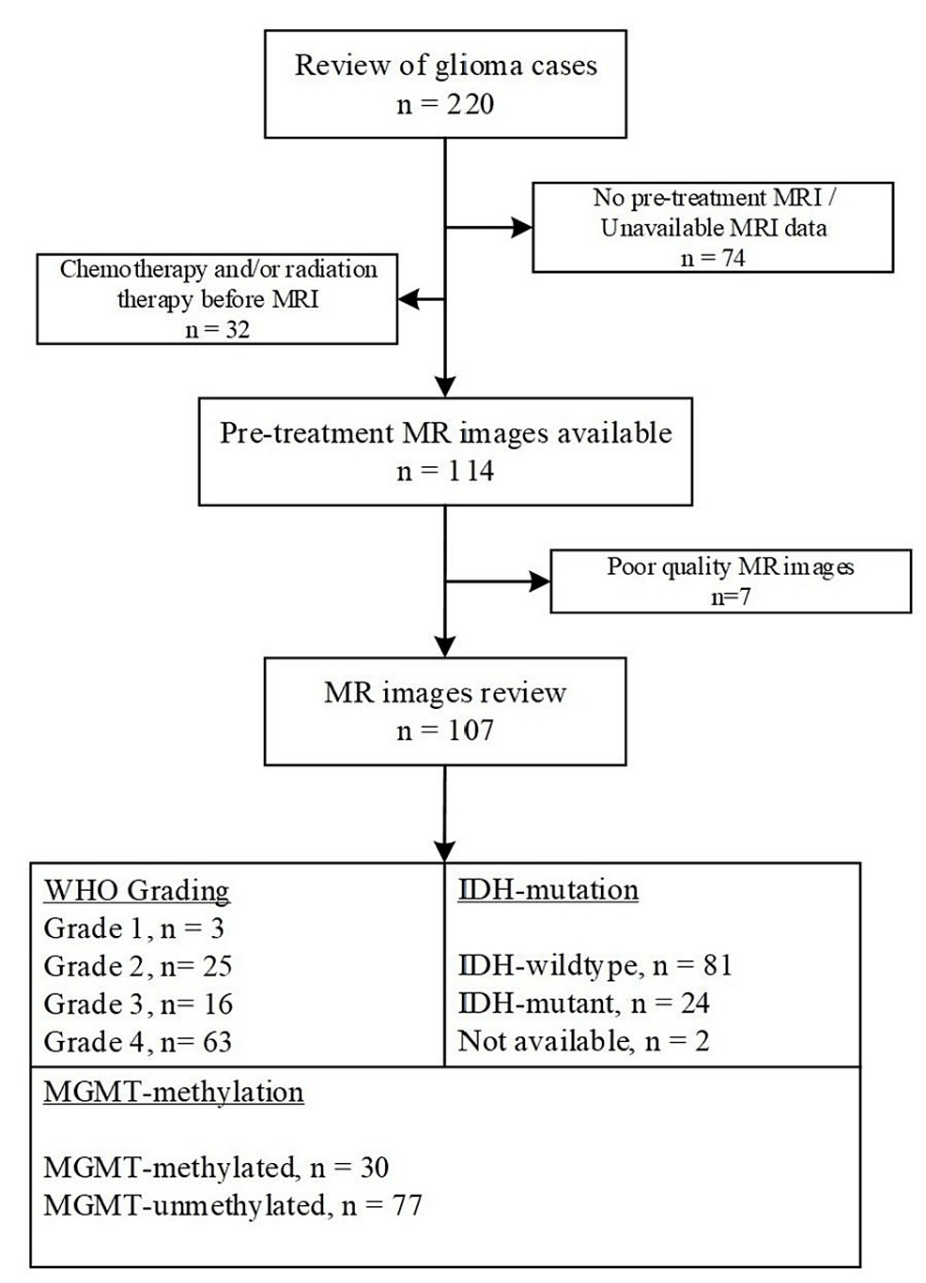
Subject selection process. IDH, isocitrate dehydrogenase; MGMT, O6-methylguanine-DNA methyltransferase; MRI, magnetic resonance imaging; WHO, World Health Organization

Between November 2017 and November 2022, our institution treated 220 glioma patients. A detailed outline of the patient selection process is presented in Figure 1. Medical records of 107 patients who met the inclusion and exclusion criteria were obtained from our institutional records. Imaging data were retrieved from the institution's picture archiving and communication system.

MRI protocol

All patients underwent MRI using either a 1.5-T Philips Multiva (Philips HealthCare, Best, Netherlands) or a 3T Siemens Skyra (Siemens, Erlangen, Germany). The diagnostic protocol involved a contrast-enhanced 3D volumetric spin echo T1-weighted imaging sequence after administration of the intravenous Gadolinium-based contrast agent, Gadovist (Bayer AG, Germany) - dosed at 0.1 mmol/kg of body weight. Detailed parameters for the MR sequences can be found in Table 1.

**Table 1:** MRI parameters used in two MR systems. FLAIR, fluid-attenuated inversion recovery; T1-WI, T1-weighted images; T2-WI, T2-weighted images; DWI, diffusion-weighted images; ADC, apparent diffusion coefficient

MR sequences	Philips Multiva 1.5 T	Siemens Skyra 3 T
Axial T2-FLAIR		
Slice thickness	5 mm	4.5 mm
Pixel/voxel size	0.89 mm x 0.89 mm	0.85 mm x 0.85 mm
Time echo	140 ms	85 ms
Time repetition	9,000 ms	8,000 ms
Inversion time	2,700 ms	2,372 ms
Acquisition matrix	256 x 256	256 x 256
Axial T1-WI		
Slice thickness	5 mm	4.5 mm
Pixel/voxel size	0.71 mm x 0.71 mm	0.68 mm x 0.68 mm
Time echo	15 ms	11 ms
Time repetition	678 ms	1300 ms
Acquisition matrix	320 x 320	320 x 320
Axial T2-WI		
Slice thickness	5 mm	4.5 mm
Pixel/voxel size	0.34 mm x 0.34 mm	0.49 mm x 0.49 mm
Time echo	120 ms	111 ms
Time repetition	4,000 ms	5,000 ms
Acquisition matrix	672 x 672	448 x 392
3D T1-WI post-contrast administration		
Slice thickness	1.2 mm	0.9 mm
Pixel/voxel size	0.7 x 0.7 mm	0.89 x 0.89 mm
Time echo	9.3 ms	11 ms
Time repetition	400 ms	700 ms
Acquisition matrix	352 x 352	256 x 256
Axial DWI and ADC		
Slice thickness	5 mm	4 mm
Pixel/voxel size	0.7 mm x 0.7 mm	1.72 mm x 1.72 mm
Time echo	72 ms	59 ms
Time repetition	3,500 ms	5,870 ms
Acquisition matrix	336 x 336	128 x 128
*b*-value	0 and 1,000	0 and 1,000

VASARI assessment

Two radiologists, each with five years of experience in brain glioma cases, evaluated the visual radiological features. This evaluation took place in a well-lit room using diagnostic monitors and the Osirix Dicom Viewer software version 8.5 (Pixmeo, Switzerland). The radiologists were blinded to essential patient data, including histopathological findings and molecular information. Disagreements were settled through discussion. The VASARI criteria were used for the visual radiomic feature analysis [5]. To measure agreement between and within the radiologists, the Kappa method was employed on a random selection of research subjects. These assessments were done twice, with a four-week gap.

Pathological and molecular assessment

Tissue samples were collected either from a biopsy or previously operated tissues stored in formalin-fixed paraffin-embedded (FFPE) blocks. Each sample underwent an assessment for categorization as per the WHO 2021 Central Nervous System Tumor classification by two pathologists, unaware of other clinical assessments. The methodologies employed have been validated and documented in earlier references [9-11].

Statistical analysis

All statistical analyses were performed using the R software, version 4.3.1. The basic characteristics of patients were analyzed based on the groups of low-grade and high-grade, IDH mutation status, and MGMT methylation. Numeric data, specifically from the VASARI features f29 and f30, were *z*-normalized. Due to the high-dimensional nature of our data and the limited number of study subjects, we could not conventionally split our subjects into training and testing groups. Instead, we employed 5-fold internal cross-validation during the modeling process using machine learning approaches. In total, we utilized four machine-learning methods: Elastic-Net (from the *glmnet *package), Random Forest (*rf*), MARS (*earth*), and XGBoost (*xgbLinear*). The response variables were glioma degree (high-grade vs. low-grade), IDH mutation (mutant vs. wildtype), and MGMT methylation (methylated vs. unmethylated). All 27 VASARI features served as predictors in this predictive model formation. Systematic hyperparameter tuning was carried out using the *caretEnsemble *package with a *tuneLength *parameter set to 5. The performance of the four predictive models is presented in the form of box plots, representing AUC values, sensitivity, and specificity. Finally, variable importance was extracted from the machine-learning models.

## Results

We obtained data from 107 research subjects who met the inclusion and exclusion criteria. However, IDH mutation data was available for only 105 of these subjects. A summary of the baseline characteristics of the subjects can be found in Table 2.

**Table 2:** Subject characteristics relative to glioma grade, IDH mutation, and MGMT methylation. ^a^*n* (%); mean ± SD. ^b^Pearson's chi-squared test; Wilcoxon rank-sum test. ^*^Significant with *P*-value < 0.05. IDH, isocitrate dehydrogenase; MGMT, O6-methylguanine-DNA methyltransferase

Characteristic	Glioma grade			IDH mutation			MGMT methylation		
	High grade, *N* = 79^a^	Low grade, *N* = 28^a^	*P*-value^b^	Mutant, *N* = 24^a^	Wildtype, *N* = 81^a^	*P*-value^b^	Methylated, *N* = 30^a^	Unmethylated, *N* = 77^a^	*P*-value^b^
Sex			0.451			0.100			0.721
Male	46 (58%)	14 (50%)		17 (71%)	42 (52%)		16 (53%)	44 (57%)	
Female	33 (42%)	14 (50%)		7 (29%)	39 (48%)		14 (47%)	33 (43%)	
Age at diagnosis	50.35 ± 13.90	33.32 ± 12.44	0.000^*^	37.42 ± 10.54	48.40 ± 15.99	0.000^*^	48.30 ± 11.40	44.96 ± 16.71	0.517

The kappa value for inter-observer consistency varied between 0.714 and 0.831, signifying a good level of consensus among different evaluators for all VASARI features. Regarding intra-observer consistency, assessments were made with a four-week gap, resulting in a kappa value of 0.91. This value is categorized as nearly perfect agreement, emphasizing the high level of consistency by individual observers at different assessment times.

Glioma grade

**Figure 2:**
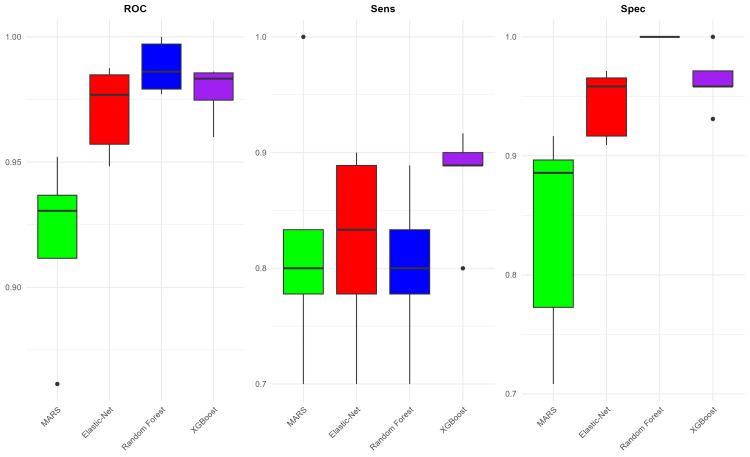
Glioma grade model performance. Comparison of model performances for tumor grade prediction based on VASARI features. This figure presents box plots illustrating the AUC, sensitivity, and specificity values for four machine-learning models after undergoing fivefold cross-validation. AUC, area under the ROC Curve; MARS, multivariate adaptive regression spline; ROC, receiver operating characteristic curve; VASARI, Visually AcceSAble Rembrandt Images; XGBoost, eXtreme Gradient Boosting

**Figure 3:**
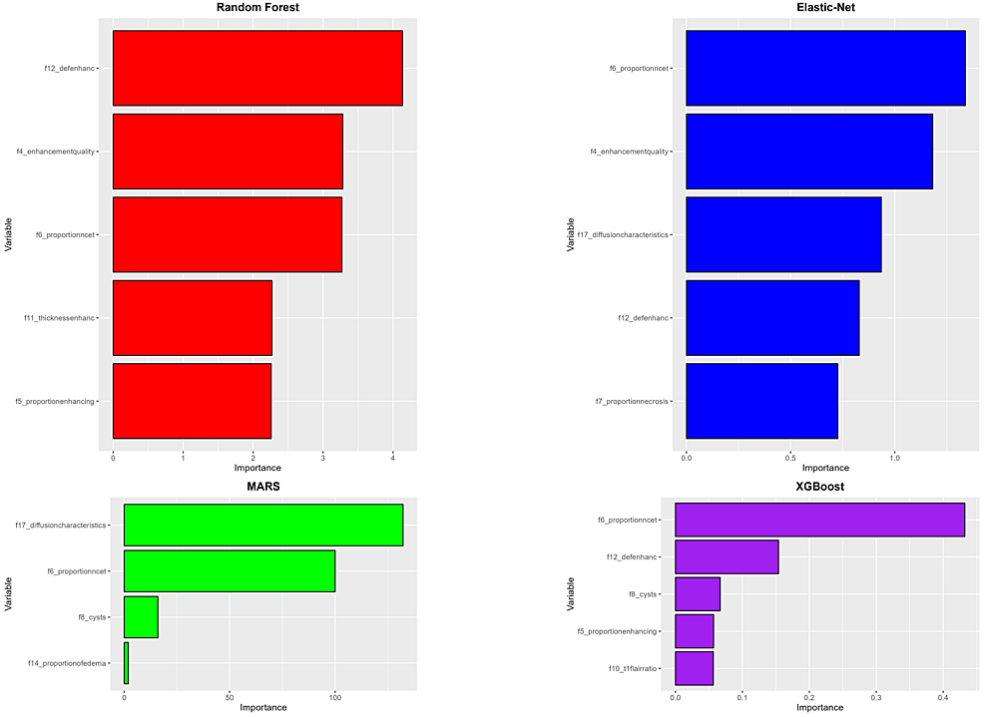
Variable importance (grade). Importance ranking of VASARI predictor variables across machine-learning models. This figure displays the most important VASARI variables contributing to glioma grade prediction in four machine-learning models. MARS, multivariate adaptive regression spline; VASARI, Visually AcceSAble Rembrandt Images; XGBoost, eXtreme Gradient Boosting

The performances of these models are presented as box plots indicating their AUC, sensitivity, and specificity values after undergoing fivefold cross-validation (Figure 2). Random Forest achieved the highest mean AUC of 0.988, while the MARS model had the lowest performance with an AUC of 0.918. The highest average sensitivity was achieved by the XGBoost model, standing at 0.879, while the lowest sensitivity was observed in the MARS and Random Forest model at 0.800. In terms of specificity, the Random Forest model excelled with a perfect score of 1.000, whereas the MARS model registered the lowest average specificity at 0.836. We can also observe which VASARI predictor variables have the highest importance in the machine learning model. Figure 3 shows the most important variables in each of those models. The VASARI feature f6 (proportion of non-enhancing tumor) appears in all four models, while f12 (definition of the enhancing margin) appears in three of the models. This indicates the consistency of these features as predictors of tumor grade.

IDH mutation

**Figure 4:**
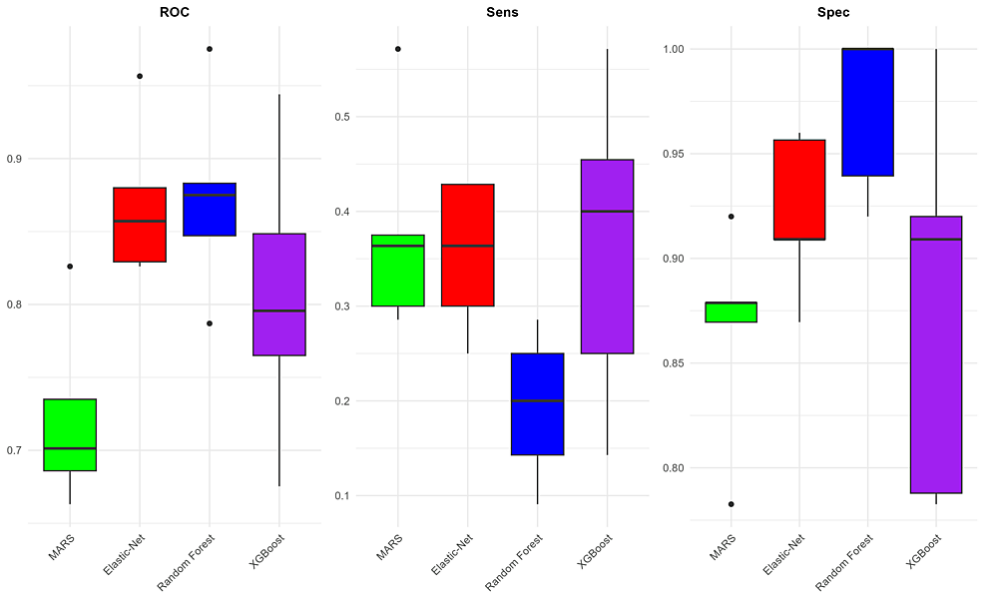
Glioma IDH model performance. Comparison of model performances for IDH status prediction based on VASARI features. This figure presents box plots illustrating the AUC, sensitivity, and specificity values for four machine-learning models after undergoing fivefold cross-validation. AUC, area under the ROC curve; IDH, isocitrate dehydrogenase; MARS, multivariate adaptive regression spline; ROC, receiver operating characteristic; VASARI, Visually AcceSAble Rembrandt Images; XGBoost, eXtreme Gradient Boosting

**Figure 5:**
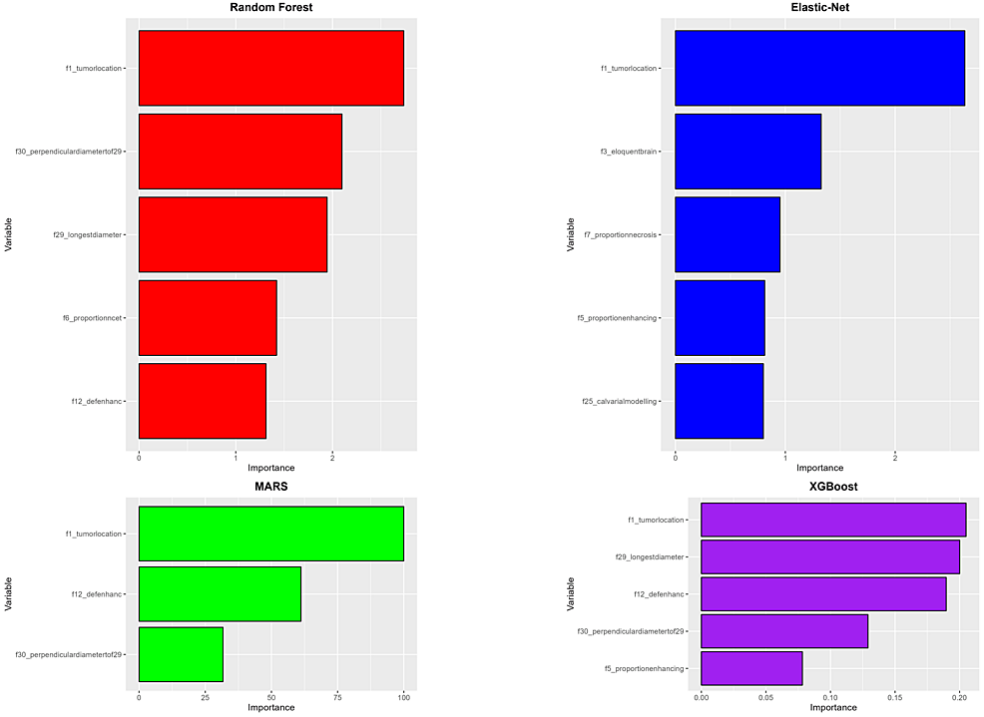
Variable importance (IDH). Importance ranking of VASARI predictor variables across machine learning models. This figure displays the most important VASARI variables contributing to glioma IDH status prediction in four machine-learning models. IDH, isocitrate dehydrogenase; MARS, multivariate adaptive regression spline; VASARI, Visually AcceSAble Rembrandt Images; XGBoost, eXtreme Gradient Boosting

The highest average AUC was achieved by the Random Forest model with a score of 0.873, while the MARS model recorded the lowest AUC with an average of 0.722. Regarding sensitivity, the best-performing MARS model only reached a sensitivity of 0.379. The highest specificity was observed in the Random Forest model with an impressive average of 0.972, whereas the lowest specificity was in the MARS model, at 0.866 (Figure 4). The most significant variables in all four models are shown in Figure 5. The variable f1 (tumor location) emerged in all four models, underscoring its consistent importance in predicting IDH mutation status. The variables f12 (definition of the enhancing margin) and f30 (perpendicular diameter to f29) appeared in three out of the four models, emphasizing the significance of glioma enhancement characteristics and size in predicting IDH mutation status.

MGMT methylation

**Figure 6:**
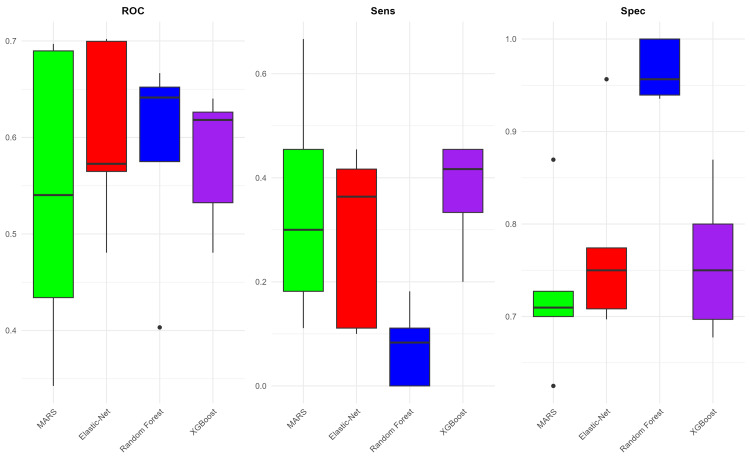
Glioma MGMT model performance. Comparison of model performances for MGMT methylation prediction based on VASARI features. This figure presents box plots illustrating the AUC, sensitivity, and specificity values for four machine-learning models after undergoing fivefold cross-validation. AUC, area under the ROC curve; MARS, multivariate adaptive regression spline; MGMT, O6-methylguanine-DNA methyltransferase; ROC, receiver operating characteristic; VASARI, Visually AcceSAble Rembrandt Images; XGBoost, eXtreme Gradient Boosting

**Figure 7:**
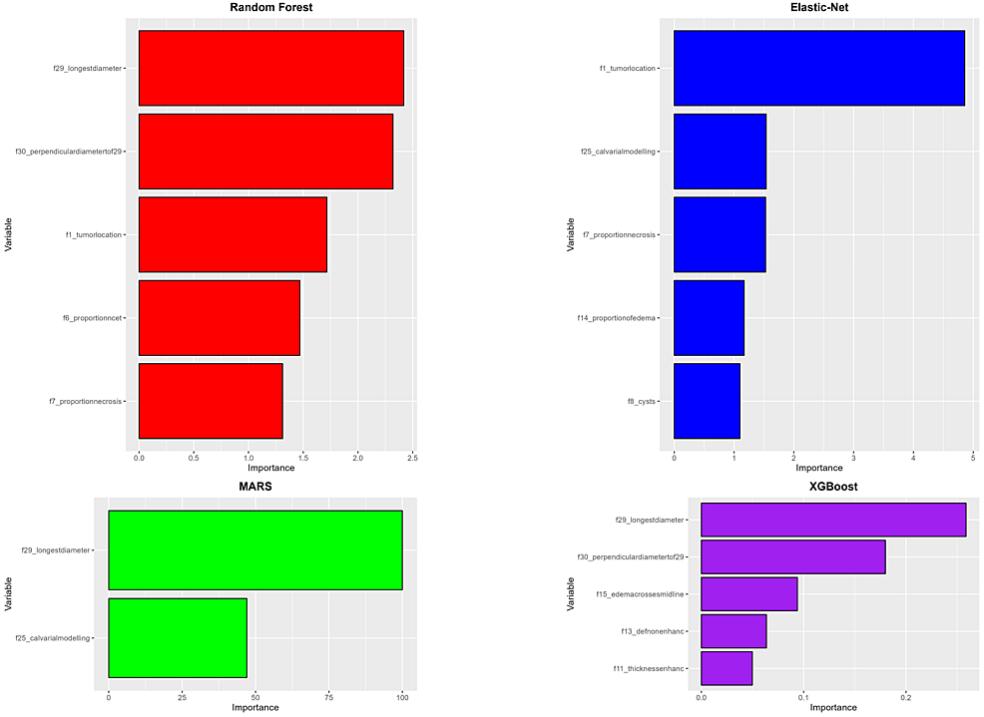
Variable importance (MGMT). Importance of ranking of VASARI predictor variables across machine-learning models. This figure displays the most important VASARI variables contributing to glioma MGMT methylation prediction in four machine-learning models. MARS, multivariate adaptive regression spline; MGMT, O6-methylguanine-DNA methyltransferase; VASARI, Visually AcceSAble Rembrandt Images; XGBoost, eXtreme Gradient Boosting

The performance of the models is displayed in Figure 6. The best average AUC was for the Elastic-Net model, and the lowest was for MARS, with respective average AUCs of 0.604 and 0.541. The sensitivity of these four machine learning models was not particularly strong in predicting MGMT status, with the best average sensitivity found in the XGBoost model (0.372) and the lowest in the Elastic-Net model (0.289). However, the models' specificity was quite good, with the best average result in the Random Forest model (0.966) and the lowest in the MARS model (0.726). The VASARI variable that played a significant role is f29 (longest diameter), which appeared in three out of the four models. Several other variables also appeared in two out of the four models, namely f1 (tumor location), f7 (proportion of necrosis), f25 (calvarial modeling), and f30 (perpendicular diameter to f29), as shown in Figure 7.

## Discussion

From our study subjects, there was a statistically significant difference in age at diagnosis between high-grade glioma and low-grade glioma patients (50.35 ± 13.90 vs. 33.32 ± 12.44, *P* = 0.000). Furthermore, we also found that the average age of glioma patients with IDH-mutant was significantly younger compared to glioma patients with IDH-wildtype (37.42 ± 10.54 vs. 48.40 ± 15.99, *P* = 0.000). However, no significant difference in age at diagnosis was found between glioma patients with MGMT-methylated and MGMT-unmethylated (48.30 ± 11.40 vs. 44.96 ± 16.71, *P* = 0.517). These findings support many previous studies that found that the degree of glioma tends to increase with age at diagnosis [12-15]. The cause of this phenomenon is not yet known for certain, but some theories suggest the influence of immune system changes with age, cell aging, and progression from low-grade glioma in younger ages to high-grade glioma in older ages [13,14]. IDH-mutant is also commonly found in younger ages, especially in secondary glioblastomas and low-grade gliomas [15,16]. Both types of gliomas tend to appear at a younger age, so, in terms of frequency, IDH-mutant glioma will also be found more often in younger age groups.

In this study, we employed four distinct machine-learning models to classify glioma grade, IDH mutation, and MGMT methylation based on VASARI features. These models provide a mix of linear and nonlinear, as well as parametric and nonparametric methods, offering the potential to capture different types of relationships in the data [17-19]. Using multiple models also provides an opportunity to cross-validate findings across different methodologies, increasing the robustness of study conclusions.

Four machine learning models demonstrated comparable performance in predicting glioma grades, with an average AUC ranging from 0.918 to 0.988. However, it's essential to emphasize sensitivity performance since the models were trained to detect high-grade gliomas, which have a worse prognosis than low-grade gliomas. Based on its sensitivity value, XGBoost exhibited the best sensitivity in detecting glioma grades, at 87.9%. The predictor variables that appeared most frequently in all four models were f6 and f12. These findings are partly consistent with some previous studies. One study reported that VASARI features, such as enhancement quality and proportion enhancement, were significantly higher in high-grade gliomas compared to low-grade gliomas [20]. The reason enhancement characteristics can be distinctive features of glioma grades is likely related to a more serious breakdown of the blood-brain barrier in high-grade glioma. Furthermore, high-grade gliomas have a higher degree of neovascularity, albeit with inferior blood vessel quality, often resulting in the leakage of the contrast agent appearing as an enhancement on MRI examinations [20].

Predicting IDH mutation status and MGMT promoter methylation in glioma patients is of significant clinical importance as these molecular markers play a crucial role in determining prognosis and guiding treatment decisions. IDH-mutant gliomas have been shown to have a more favorable prognosis compared to IDH-wildtype gliomas [21]. In our study, the AUC of the four machine learning models to predict IDH mutation was reasonably good (ranging from 0.722 to 0.873), with Random Forest having the highest AUC. The specificity of these models was also good, with Random Forest producing the best results (97.2%). Unfortunately, these four models did not demonstrate adequate sensitivity in detecting IDH-mutant vs. IDH-wildtype. The machine learning models we developed assume that IDH-mutant is class 0 and IDH-wildtype is class 1, which is also the prediction target. This suggests a considerable portion of IDH-wildtypes may be misclassified as mutants, which could lead to suboptimal clinical decisions if one solely relies on the model's predictions. Misclassifying IDH-wildtype cases as mutants might cause patients not to receive the most appropriate care tailored to their mutation status.

The three main variables to predict IDH mutation status in our study were f1, f12, and f30. One study found that VASARI features, including the proportion of necrosis and lesion size, were associated with IDH1 mutation status in gliomas [22]. Another study yielded results somewhat similar to ours, where IDH-mutant gliomas are often located in the frontal lobes, have a larger proportion of non-enhancing tumors, and display a more diffuse growth pattern as represented in T1/FLAIR morphology [23]. IDH-mutant gliomas may show less or no contrast enhancement, while the presence and proportion of necrosis might differ based on IDH status [24]. Another researcher found that IDH-mutant gliomas tend to have a more defined margin compared to wildtype [25].

Methylation of the MGMT promoter leads to the silencing of the gene and reduced expression of the MGMT protein. This makes tumor cells more sensitive to alkylating agents [26]. Glioma patients with MGMT promoter methylation have been shown to respond better to alkylating agent chemotherapy, such as temozolomide, and have improved overall survival compared to those without MGMT promoter methylation [27].

In our study, the models failed to achieve a satisfactory AUC to predict MGMT methylation status. The best result was observed in the Elastic-Net model (AUC = 0.604). The sensitivity of these models was also subpar, with the highest sensitivity of 37.2% observed in the XGBoost model. However, the specificity performance was generally better, with the Random Forest model demonstrating a specificity of 96.6%. Despite this, we anticipated that the models would effectively predict the presence of unmethylation in MGMT.

The tumor size (f29) appeared to be the only predictor variable playing a significant role in these models, featuring in three out of the four models. Few studies have discussed the role of VASARI features in distinguishing between gliomas with methylated and unmethylated MGMT. One study found that MRI features, such as the proportion of enhancing and non-enhancing tumors, the amount of restricted diffusion, and the proportion of edema, significantly varied between gliomas with methylated MGMT versus unmethylated MGMT. However, that study only analyzed VASARI variables individually using conventional analysis and did not consider interactions among VASARI variables [28].

The XGBoost algorithm exhibited the best overall performance in predicting glioma grade, IDH mutation status, and MGMT methylation, demonstrating the most balanced score for AUC, sensitivity, and specificity values. XGBoost has gained considerable attention in the field of medical imaging for its robustness and high predictive accuracy. For instance, it has been employed in the automated diagnosis of various medical conditions, ranging from detecting lung nodules in chest X-rays to identifying lesions in mammograms and Alzheimer’s disease, superior to Random Forest and support vector machine [29]. This superior performance can be attributed to XGBoost's gradient boosting framework, which sequentially constructs an ensemble of decision trees optimized for prediction accuracy. The algorithm also incorporates both L1 (Lasso) and L2 (Ridge) regularization techniques, enhancing its resilience against overfitting critical factors given the study's relatively small sample size. Its ability to manage both linear and non-linear relationships in the data makes it particularly adept at capturing the intricate patterns associated with glioma grade and molecular markers, such as IDH mutation status.

**Figure 8:**
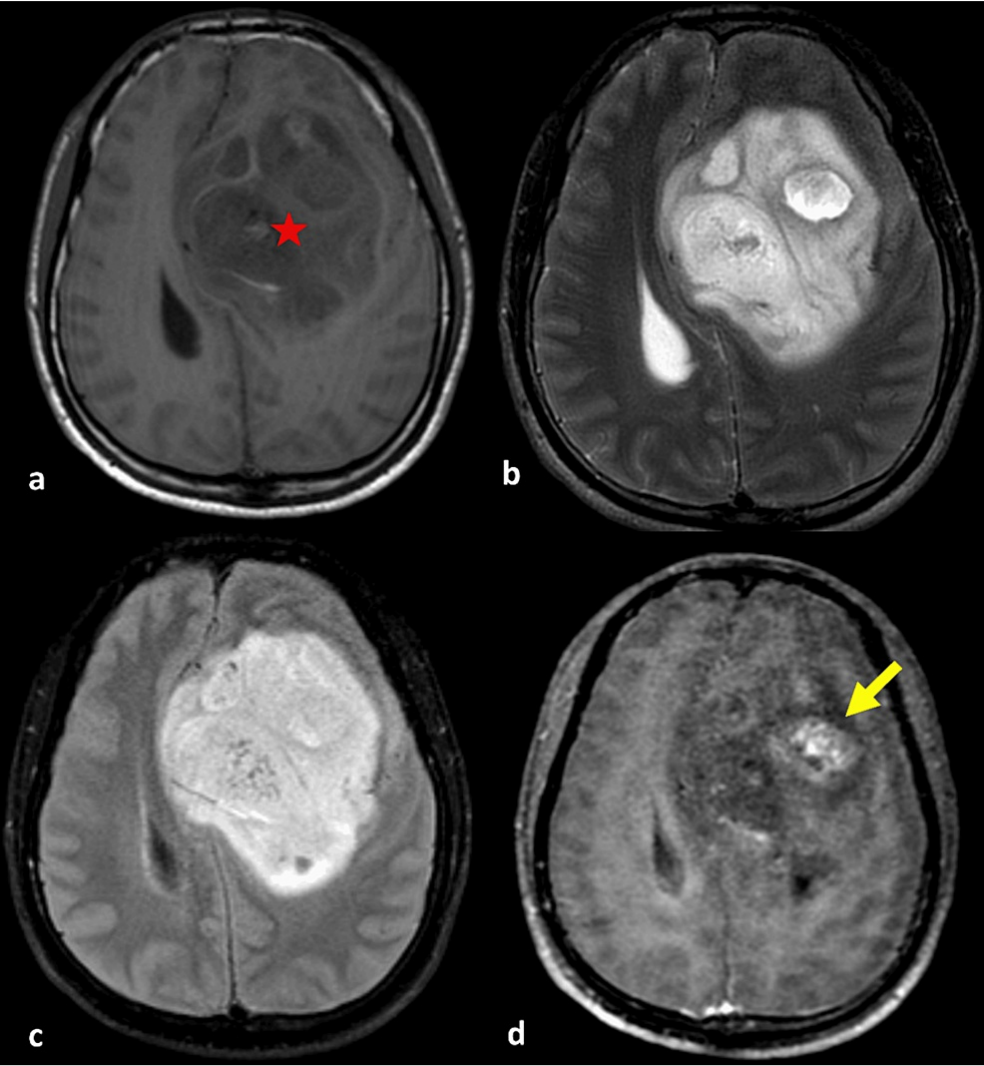
A 29-year-old male with a heterogeneous intra-axial mass centered in the left frontal lobe. (a) Axial T1-weighted, (b) T2-weighted, (c) FLAIR, and (d) post-contrast administration T1-weighted MR images showed solid non-enhancing area (f6), comprising 68%-100% of the total FLAIR abnormality area (red star). The enhancing component has relatively well-defined boundaries (f12) and marked enhancement quality (f4) (yellow arrow). While this lesion appearance strongly suggests a low-grade glioma, histopathological and molecular results confirm this case to be an astrocytoma, IDH-mutant, CNS WHO grade 4. IDH, isocitrate dehydrogenase; FLAIR, fluid-attenuated inversion recovery; MR, magnetic resonance; WHO, World Health Organization; CNS, central nervous system

**Figure 9:**
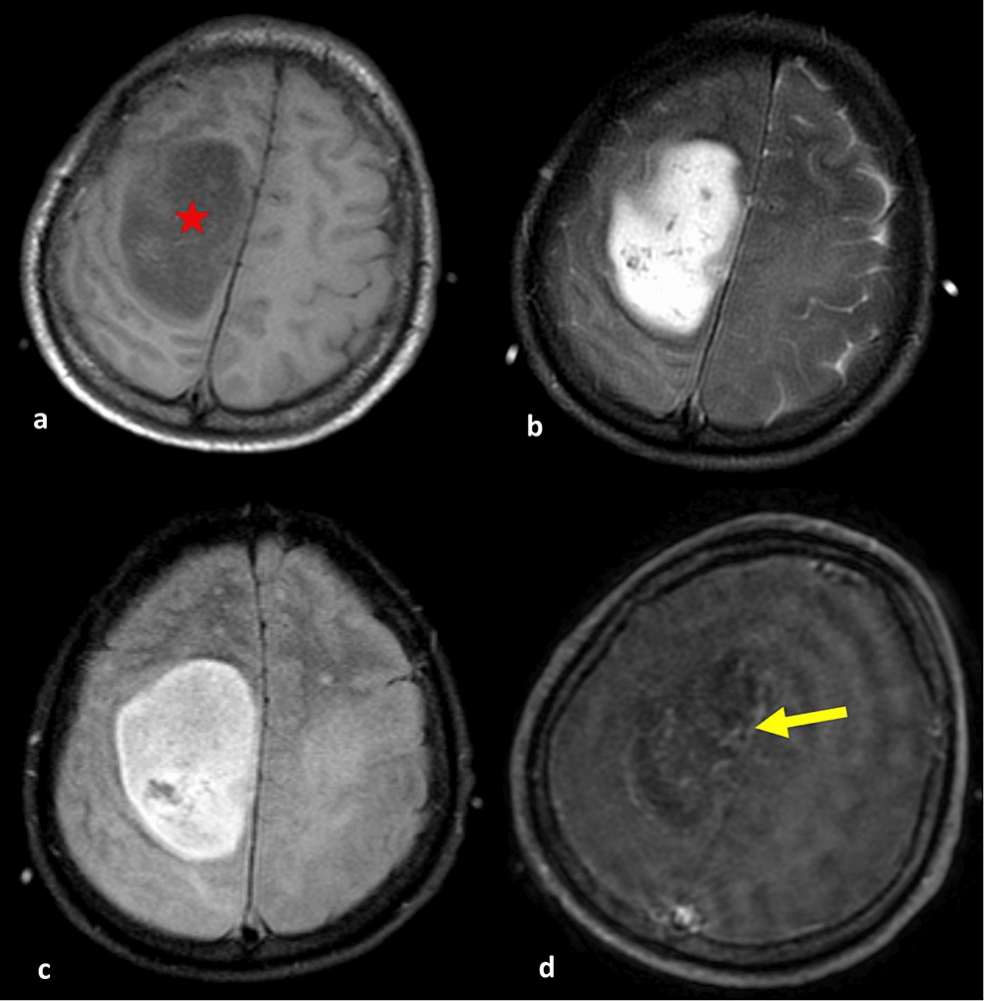
A 55-year-old male presenting with an intra-axial mass. (a) Axial T1-weighted, (b) T2-weighted, (c) FLAIR, and (d) post-contrast T1-weighted MR images showed the tumor is located in the right frontal lobe (f1) (red star) and exhibits ill-defined boundaries in the enhancing tumor region (f12) (yellow arrow). There is an absence of perifocal edema, extensive necrotic areas, or nodular solid-enhancing regions. Although the morphological features of this lesion suggest a low-grade diffuse astrocytoma, molecular examination reveals an IDH-wildtype. Consequently, according to the WHO CNS 2021 classification, this tumor is categorized as an astrocytoma, IDH-wildtype, CNS WHO grade 4. IDH, isocitrate dehydrogenase; FLAIR, fluid-attenuated inversion recovery; MR, magnetic resonance; WHO, World Health Organization; CNS, central nervous system

The combination of IDH mutation status and MGMT promoter methylation provides even more valuable information for predicting prognosis and guiding treatment decisions in glioma patients. IDH-mutant gliomas with MGMT promoter methylation have been associated with a more favorable prognosis compared to IDH-wildtype gliomas with MGMT promoter methylation [30]. Therefore, the combination of IDH mutation status and MGMT promoter methylation can help stratify glioma patients into different prognostic and treatment response groups. In Figure 8 and Figure 9, we display two examples of patients from our study along with their respective VASARI feature assessments.

Study limitations

Our study had several limitations that should be addressed in future research. First, the relatively small cohort size may introduce variability and reduce the statistical power of our findings. To overcome this, future studies should include larger, multi-institutional datasets to improve the generalizability of the results. Second, the lack of an external validation dataset limits the ability to confirm the robustness of our models. Incorporating external validation with independent cohorts would strengthen the validity of our findings. Third, there is a potential risk of overfitting due to the limited number of study subjects and the high-dimensional nature of the data. Employing advanced regularization techniques and more sophisticated machine learning algorithms can help mitigate this risk. Additionally, further research should explore the interactions among VASARI features to enhance model accuracy and predictive power.

## Conclusions

The most balanced overall performance in predicting glioma grade, IDH mutation status, and MGMT methylation status was achieved by the XGBoost method. For the glioma grade, it had an AUC of 0.978, a sensitivity of 0.879, and a specificity of 0.964. For IDH mutation status, the AUC was 0.806, sensitivity was 0.364, and specificity was 0.880. Finally, for MGMT methylation, XGBoost delivered an AUC of 0.580, sensitivity of 0.372, and specificity of 0.759. When predicting tumor grade, the predictors f6 (proportion of non-enhancing) and f12 (definition of the enhancing margin) are the most important. For IDH mutation status, predictors f1 (tumor location), f12 (definition of the enhancing margin), and f30 (perpendicular diameter to f29) are the most frequent predictors. For MGMT methylation, the most influential predictor is f29 (longest diameter).

In conclusion, the machine-learning approach to VASARI features exhibits excellent overall performance in predicting glioma grade. However, in terms of predicting IDH mutations and MGMT methylation, sensitivity has yet to achieve satisfactory results. Further research with larger cohorts, improved study designs, and deeper utilization of artificial intelligence is imperative to enhance our understanding of gliomas, ultimately improving the outcomes of glioma patient management.
